# Genetic Diversity in *Lens* Species Revealed by EST and Genomic Simple Sequence Repeat Analysis

**DOI:** 10.1371/journal.pone.0138101

**Published:** 2015-09-18

**Authors:** Harsh Kumar Dikshit, Akanksha Singh, Dharmendra Singh, Muraleedhar Sidaram Aski, Prapti Prakash, Neelu Jain, Suresh Meena, Shiv Kumar, Ashutosh Sarker

**Affiliations:** 1 Division of Genetics, Indian Agricultural Research Institute, New Delhi 110012, India; 2 ICARDA, B.P. 6299, Station Experiment, INRA-Quich, Rue Hafiane Cherkaoui. Agdal, Rabat-Institutes, Rabat, Morocco; 3 ICARDA, South Asia and China Regional Program, CGIAR Block, NASC Complex, New Delhi-110012, India; National Institute of Plant Genome Research, INDIA

## Abstract

Low productivity of *pilosae* type lentils grown in South Asia is attributed to narrow genetic base of the released cultivars which results in susceptibility to biotic and abiotic stresses. For enhancement of productivity and production, broadening of genetic base is essentially required. The genetic base of released cultivars can be broadened by using diverse types including bold seeded and early maturing lentils from Mediterranean region and related wild species. Genetic diversity in eighty six accessions of three species of genus *Lens* was assessed based on twelve genomic and thirty one EST-SSR markers. The evaluated set of genotypes included diverse lentil varieties and advanced breeding lines from Indian programme, two early maturing ICARDA lines and five related wild subspecies/species endemic to the Mediterranean region. Genomic SSRs exhibited higher polymorphism in comparison to EST SSRs. GLLC 598 produced 5 alleles with highest gene diversity value of 0.80. Among the studied subspecies/species 43 SSRs detected maximum number of alleles in *L*. *orientalis*. Based on Nei’s genetic distance cultivated lentil *L*. *culinaris* subsp. *culinaris* was found to be close to its wild progenitor *L*. *culinaris* subsp. *orientalis*. The Prichard’s structure of 86 genotypes distinguished different subspecies/species. Higher variability was recorded among individuals within population than among populations.

## Introduction

The cultivated lentil (*Lens culinaris* subsp. *culinaris*) is annual, diploid (2n = 2x = 14) with genome size of ~4 Gbp [1]. This crop was domesticated in Near East approximately 10,000 years ago [2]. The scientific name *Lens culinaris* was given by Medikus in 1787 [3]. The genus *Lens* includes both cultivated and wild forms distributed in West Asia and North Africa. However, wild forms are confined to Mediterranean region. The genus *Lens* comprises seven taxa in four species namely; *L*. *culinaris* Medikus [with subspecies *culinaris*, *orientalis* (Boiss.) Ponert, *tomentosus* (Ladizinsky) Fergusan, Maxted, Slageren & Robertson and *odemensis* (Ladizinsky) Fergusan, Maxted, Slageren & Robertson], *L*. *ervoides* (Brign.) Grande, *L*. *nigricans* (M.Bieb.) Godron and *L*. *lamottei* Czefr [4]. The cultivated lentils were classified into two groups by Barulina [5] based on seed size: microsperma / small seeded type (seed diameter less than 6 mm) and macrosperma / large seeded types (seed diameter over 6 mm). The spread of lentil to different regions from Mediterranean region led to evolution of six geographical groups, on the basis of morphological, physiological and functional variation [3]. South Asian lentils are traditionally *pilosae* type exhibiting precocity in flowering and maturity, low biomass, small seed, short or rudimentary tendrils and pubescence on foliage. This type of germplasm has shown narrow genetic base, hence introgression of genes from the Mediterranean material has been recommended [6–9] for broadening the genetic base.

Globally the crop is grown in 4.34 million hectare with production of 4.95 million ton [10]. Lentil is traditionally grown in Asia, Mediterranean region and has active diffusion in America. The main lentil producing countries are Canada, India, Turkey, Australia, USA, Nepal, China, Ethiopia, Syria and Bangladesh. Lentil grains are rich source of nutritious protein, carbohydrates, minerals and vitamins and are mainly consumed as dhal and fried snacks in Indian subcontinent and as soup and muchadara in Mediterranean region and also as sprout in other regions. Lentil straw is valued animal feed particularly in Mediterranean region. In India lentil occupied 1.89 million hectares with production of 1.13 million tonnes [10]. It is mainly grown in rain fed areas of Madhya Pradesh, Uttar Pradesh, Bihar and West Bengal. The productivity of lentil in India is around 600 kg/ha in comparison to global average of about 1000 kg/ha. The reasons for low productivity include short growing period, narrow genetic base of released cultivars, biotic stresses like wilt, rust and stemphillium blight and abiotic stresses like drought and heat. Despite systematic and continuous progress of conventional breeding programs productivity of lentil have exhibited limited progress, due to high environmental effects, genotype x environment interactions and repeated use of few lines in breeding programmes [11]. The lentil domestication led to a loss of genetic diversity of approximately 40% [12]. Improvement of crop productivity depends on the genetic variation and most studies have indicated that 50% increase in crop productivity is due to genetic improvement [13]. The pedigree analysis of Indian lentil varieties confirmed their narrow genetic base [14]. For improving the productivity and broadening the genetic base, use of Mediterranean land races and crossable wild subspecies is desired.

The knowledge of exploitable genetic diversity is essential to enhance the productivity of cultivated lentil and to insulate it against biotic and abiotic stresses. The initial diversity studies in lentil relied heavily on morphological and agronomical traits. The major limitation of morphological and agronomical traits include poor repeatability, low levels of polymorphism, phenotypic plasticity etc. The advent of molecular markers differing in degree of polymorphism, locus specificity and abundance in genome enhanced the possibilities for the diversity assessment of plant genetic resources intended to be used for crop improvement [15] [16]. The efficient diversity assessment essentially requires neutral, co dominant and polymorphic molecular markers distributed throughout the genome [17]. Isozyme markers and DNA markers like restriction fragment length polymorphisms (RFLPs) and microsatellites or simple sequence repeats (SSRs) fulfill the requirements. Isozymes were used to study genetic variation in cultivated lentil [18–20]. However isozymes failed in distinguishing closely related genotypes. RAPD, RFLP and AFLP markers were utilized by several workers [21–26] to study genetic diversity in *Lens* species. Alo *et al*. [10] used recent techniques of comparative genomics to characterize different *Lens* species. The conserved primers (CPs) based on *Medicago truncatula* EST sequences flanking one or more introns were utilized. The poor availability of genomic resources has hindered the progress of molecular characterization, mapping and tagging of important genes with molecular marker. Few reports are available on development of genomic SSR markers [27–29]. Kaur *et al*. [30] and Jain *et al*. [31] reported EST SSRs and studied their polymorphism within and between *Lens* species. EST-SSRs can be rapidly developed at low cost. Varshney *et al*.[32] have reported utility of EST SSRs in comparative and evolutionary studies.

Breeding for improved cultivars of lentil is difficult due to narrow genetic base and high G x E interactions. New technologies can assist in detection of association of agronomic traits with genetic markers and genetic maps can aid in tuning the breeding programme. Molecular markers can enhance the efficiency of selection. Microsatellites or simple sequence repeats (SSRs) are randomly distributed short stretches of DNA consisting of tandemely repeated units of 1–6 base pairs. SSRs are highly polymorphic, multi allelic, co-dominant, relatively abundant and simple to detect by PCR [33–36]. Microsatellite markers have been utilized for various applications such as genetic diversity analysis, variety identification, and phylogenetic relationships, construction of linkage maps, mapping agriculturally and economically useful genes and marker-assisted selection [32], [37–41]. Enriched genomic libraries and random genomic sequences derived from inter-genic DNA region are used to develop genomic SSRs [42–43]. The procedure is time-consuming, expensive and laborious. EST-SSRs target transcribed region of genome and have potential for linkage with loci for agronomic traits [43]. EST-SSRs with high expression of diversity between wild and cultivated accessions can be utilized in introgression breeding to identify interspecific hybrid for transfer of genes of agricultural importance (biotic and abiotic stresses) from wild to cultivated species. In addition to this EST-SSR markers had greater cross-species amplification as they target protein-coding regions that are conserved between related species [44–45]. The present investigations were carried out to analyze and compare the utility of EST-SSRs and genomic SSRs for characterization of the genetic variability within and between different accessions of genus *Lens*.

## Materials and Methods

### Plant material

The plant material studied comprised of three species of genus *Lens* (Table 1). The different accessions of wild *Lens* and cultivated Mediterranean lines were obtained from International Centre for Agricultural Research in the Dry Areas (ICARDA), Aleppo, Syria (through National Bureau of Plant Genetic Resources, New Delhi, India) and were grown under controlled conditions. ICARDA has mandate to collect and maintain the wild *lens* distributed in the Mediterranean region. The cultivated *L*. *culinaris* subsp. *culinaris* accessions studied comprised of released varieties, advanced breeding lines and Mediterranean land races including both microsperma and macrosperma types.

**Table 1:** Sources / origin of 86 accessions of different *Lens* species used in the study.

Subspecies/ species	Genotype	Source/ origin
*L*. *culinaris* subsp. *culinaris*	L830,L4076, L4147,L4595, L4602, L4603, L4618, L6183, L4704, L7903, PL01, PL04, PL05, PL06, PL07, PL08, PL406, DPL58, DPL62, IPL81, IPL321, IPL406, K75, HM 1	IARI, New Delhi, India
*L*. *culinaris* subsp. *culinaris*	ILL6002, Precoz	ICARDA, Aleppo, Syria
*L*. *culinaris* subsp. *orientalis*	IG135428, IG135443, IG135570, IG136669, IG136671, IG136673, ILWL7, ILWL11, ILWL23, ILWL24, ILWL31, ILWL50, ILWL55, ILWL70, ILWL73, ILWL81, ILWL95, ILWL96, ILWL127, ILWL131, ILWL143, ILWL147, ILWL150, ILWL152, ILWL237, ILWL242, ILWL246, ILWL342, ILWL358, ILWL378,	ICARDA, Aleppo, Syria
*L*. *culinaris* subsp. *tomentosus*	ILWL53, ILWL91, ILWL93	ICARDA, Aleppo, Syria
*L*. *culinaris* subsp. *odemensis*	IG136633, IG136655, IG136662, ILWL28, ILWL34, ILWL47	ICARDA, Aleppo, Syria
*L*. *nigricans*	IG136623, IG136626, IG136631, IG136649, IG136653, ILWL345	ICARDA, Aleppo, Syria
*L*. *ervoides*	IG62506, IG136663, IG136664, IG136665, IG136666, IG137423, IG140891, IG140893, IG140970, IG141573, ILWL44, ILWL51, ILWL52, ILWL72, ILWL77,	ICARDA, Aleppo, Syria

### Genomic DNA extraction, purification and SSR amplification

The genomic DNA was isolated from 2 gm of fresh leaf tissue following CTAB method [46]. The eighty six DNA samples representing all studied cultivated and wild accessions were quantified using spectrophotometer and diluted to 40ng/μl as preparation for polymerase chain reaction (PCR) amplification. Seventy five EST-SSRs and 27 genomic SSRs were used for study of polymorphism. The studied EST-SSRs were developed by Kaur *et al*. [30] and Jain *et al*.[31]. The genomic SSRs used were reported by Hamwieh *et al*. [27] and Saha *et al*. [47]. Thirty one EST-SSRs and twelve genomic SSRs exhibiting polymorphism were utilized for the study. The selected genomic SSRs and EST SSRs were transferable across the studied *Lens* species. Other EST-SSRs and genomic SSRs were not considered due to their monomorphic nature and / or non-specific amplification. A total reaction volume of 20 μl comprised of 10 × buffer (100 mM Tris-HCl, 500mM KCl, 15mM MgCl2, 0.01 percent gelatin); 200 μM each dNTP, 0.5 μM each of forward and reverse primers, 1U Taq DNA polymerase (PCR reagents and EST-SSR or genomic SSR primer procured from Sigma-Aldrich, Spruce Street, St. Louis, USA), ~40 ng DNA. Reactions were performed in VeritiTM (Applied Biosystems, Life Technologies, Singapore) thermal cycler using the following temperature cycle: one denaturation cycle at 94°C for 4 min followed by 30 cycles of 94°C for 1min, annealing at ranging from 51–62°C (primer specific) for 30 seconds, extension at 72°C for 1 min with final extension at 72°C for 10 min. The amplification fragments were electrophoresed for 3 h at a constant voltage of 100 V in 1X TBE buffer on 3 percent metaphor TM agarose gels (Lonza, Rockland, ME USA) and visualized using ethidium bromide staining. The products were photographed with a CCD camera attached to a gel documentation system (Syngene). 50bp DNA ladder (MBI, Fermentas, Vilnius, Lithuania) was used as molecular size marker.

### Data analysis

Only clear and unambiguous bands were scored. In each genotype, scoring was done on the basis of length polymorphism of the marker with respect to the 50bp standard DNA ladder (MBI Fermentas) and the presence (1) or absence (0) of the corresponding band among the genotypes was recorded. Polymorphism information content (PIC) was computed for each primer. PIC is an indication of band informativeness PIC = 1-ΣPi − ΣΣPiPj where ‘i’ is the total number of alleles detected for SSR marker and ‘Pi’ is the frequency of the i allele in the set of 86 genotypes investigated and j = i+1 [48]. The resolving power (Rp) of primer was calculated as Rp = ΣIb, where Ib (band informativeness) takes the value: 1 − [2 × (0.5 − p)], p being the proportion of genotype of different *Lens* subspecies / species containing that band [49]. Genetic diversity parameters viz., number of alleles (Na), observed heterozygosity (Ho), Shannon Index (I) and Nei’s genetic diversity index (He) were calculated using POPGENE v 1.31 (http://www.ualberta.ca/~fyeh). The dendrogram based on unbiased genetic distances among different species was constructed by UPGMA (Unweighted pair-group method with arithmetic average) employing POPGENE v 1.31. The data were subjected to UNJ (Unweighted Neighbour Joining method) cluster analysis followed by bootstrap analysis with 1000 permutations for total genotypes was carried out using DARwin 5.0.145 (http://darwin.cirad.fr/).

**Fig 1:**
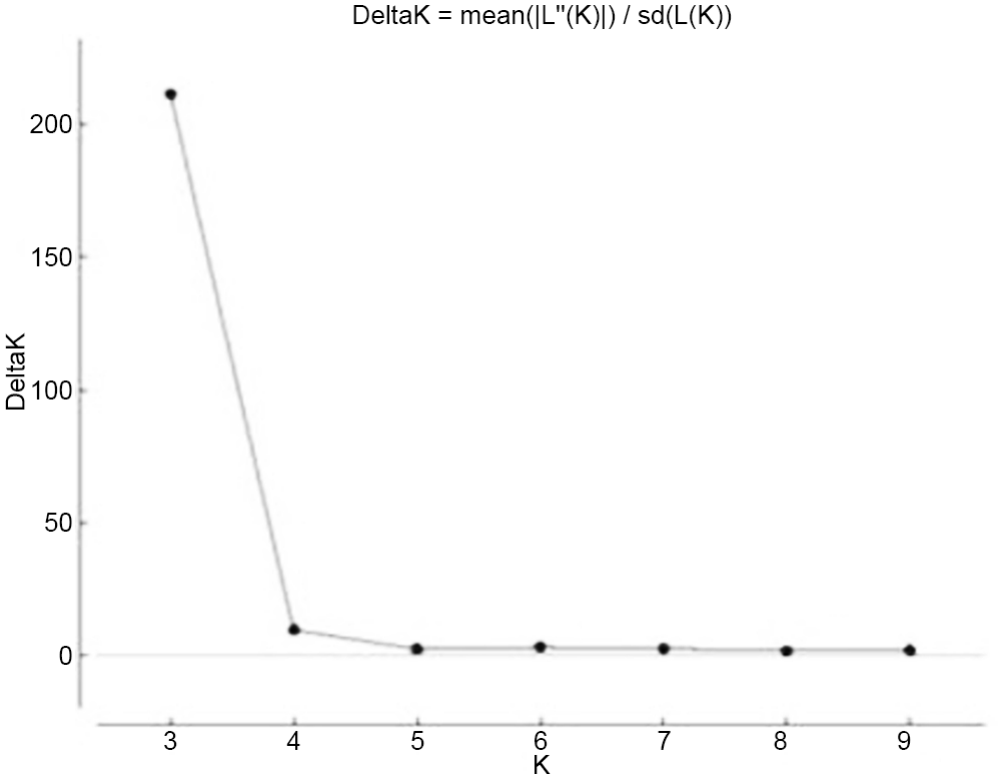
Estimation of subspecies / species of genus *Lens* using LnP(D) derived Δ k for k from 1 to 10.

Pritchard’s structure analysis Structure 2.23 (http://pritch.bsd.uchicago.edu/structure.html) was used to determine the population structure using the Bayesian clustering approach assuming prior values of k between 1 and 10 [50]. Two different analyses were carried out assuming prior population groups and without prior information on population groups. The posterior probabilities of k (i.e. the likelihood of k as a proportion of the sum of the likelihoods for different values of k) were estimated using the Markov Chain Monte Carlo Method (MCMC). The results were analysed at Burnin period length = 100 000, MCMC iterations = 100 000, and α was kept constant. Runs were repeated at least 10 times with an admixture model and correlated allele frequency to estimate the genome proportion derived from different individuals. An optimum K value was determined by employing structure harvester v 6.92 [51] that calculated delta k by plotting LnP(D) values against K. The highest plateau was observed at delta k = 3 (Fig 1) and hence the number of inferred populations were assumed to be five for further analysis. An analysis of molecular variance (AMOVA) was undertaken to partition genetic variability among and within populations using Arlequin software version 3.1 [52].

## Results

### SSR marker analysis

**Fig 2:**
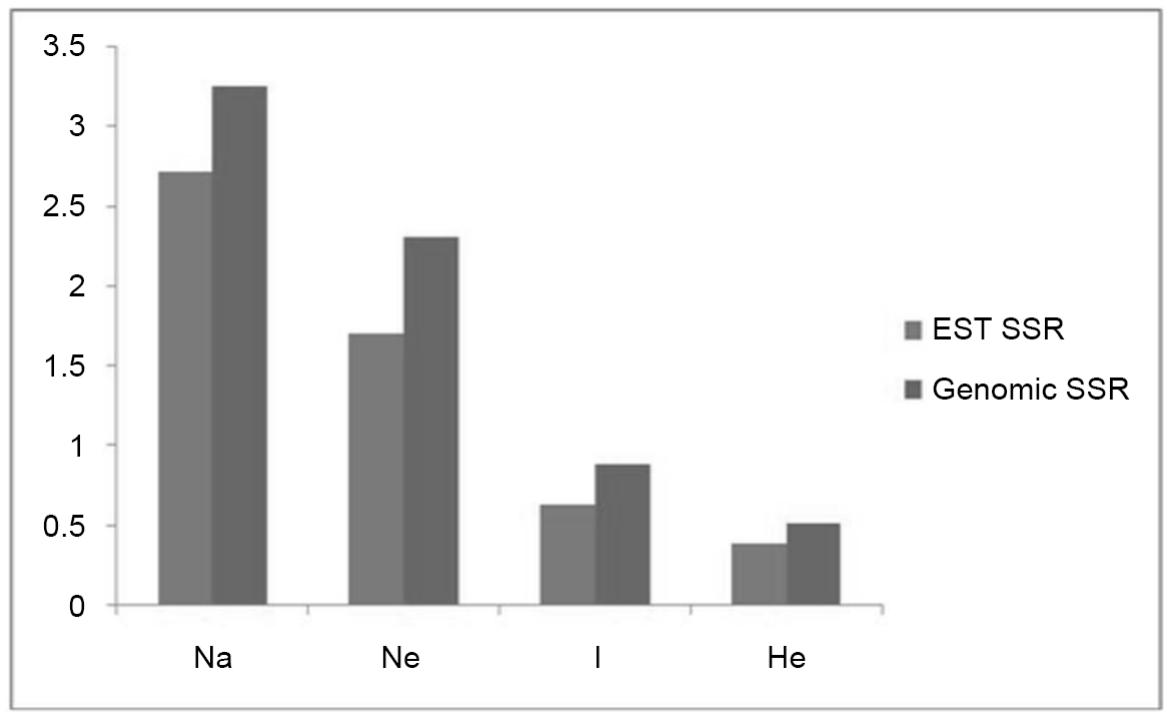
Changes in number of alleles (Na), genetic diversity (He) Shannon index (I) and Nei’s genetic diversity (He) using EST and Genomic SSR.

Thirty-one EST-SSRs and twelve genomic SSRs detected a total of 122 alleles in eighty-six genotypes (Table 2, S1 Fig) with an average of 2.89 alleles per locus. The total number of alleles detected ranged from 2 to 5 with *L*. *culinaris* subsp. *orientalis* exhibiting highest mean number of alleles (2.51). Mean maximum number of effective alleles were also present in *L*. *culinaris* subsp. *orientalis* (1.549) followed by *L*. *culinaris* subsp. *culinaris* (1.455) and *L*. *ervoides*. Shannon’s information index which is a measure of gene diversity was also higher in *L*. *culinaris* subsp. *orientalis* (0.522) and was lowest in *L*. *culinaris* subsp. *tomentosus* (0.084). The heterozygosity values ranged from 0.02 to 0.62 for EST-SSRs and from 0.24 to 0.80 for genomic SSRs among the populations (Table 2). Genomic SSRs (GLLC 106, 108, 511, 527, 538, 541, 563, 598, 609, 614, SSR 124 and SSR 154) produced higher average number of alleles, number of effective alleles, Shannon’s information index and genetic diversity in comparison to EST-SSRs (Fig 2). Genomic SSR locus GLLC598 produced maximum number of alleles (five) and highest gene diversity values (0.80) among all the loci.

**Table 2:** Tm, PIC, Rp, Na, Ne, I and He values recorded for thirty-one EST-SSRs and twelve genomic SSRs.

S.No.	Primer	Primer Sequence	Tm	PIC	Rp	Na	Ie	I	He	Reference
1	PLC5	CATTGCAGCTTATTCTCACAGC TGACCCATCCTCATCCTTAAAT	60	0.45	2	2	1.81	0.64	0.45	Neelu *et al*. 2013
2	PLC10	TGCAACAAAGGACACTAGAGGTT ATTTCTTTCTCCCTAACCAGCC	59	0.55	1.35	2	1.76	0.62	0.43	‘‘
3	PLC16	CGTTTGATCTTCTAAGCCCCTA AAGGGAAAGGATGTTTGACTTG	59	0.05	2.02	3	1.06	0.15	0.06	‘‘
4	PLC17	AAGCTGAAGGAAATCAAAGTGG TCAACACACTCCATGTTTAGAGC	59	0.48	1.7	2	1.64	0.58	0.39	‘‘
5	PLC21	AACTCGCATCCTCTTCACAACT GGACCTTTCCCTTGTAGTCACC	59	0.37	2	2	1.59	0.56	0.37	‘‘
6	PLC22	TACACTGAAGGAGATGCACTGG TAACAACAAAACACAGCTTCGC	60	0.56	2.51	4	2.46	1	0.6	‘‘
7	PLC30	TTGGTCAGGTTCTCAATCCTCT ACGGATGAACGCTTGTAAAGAA	61	0.4	2.02	3	1.68	0.7	0.41	‘‘
8	PLC35	TTGCTTCCTCCTCTTCTCACTC AGCCTCAGTACCCTCCTCTTTT	60	0.27	1.98	3	1.36	0.49	0.26	‘‘
9	PLC38	CCTGGAGAAGTCTGTGGAAGAT AGCTCTAGCATTTTGCATGTGA	59	0.37	2	2	1.59	0.56	0.37	‘‘
10	PLC40	CAACTCGCATCCTCTTCACA CAAAGGGGTTGGAGTCGTAA	60	0.35	2.02	2	1.57	0.55	0.37	‘‘
11	PLC42	AACCAATCATGGCTTCTGCT TTTCACCGTCTTTATGAACCA	60	0.55	2	3	2.22	0.87	0.55	‘‘
12	PLC46	CAAACTGGAAGATGCTGCTG TGACCCATCCTCATCCTTAAA	59	0.48	2	2	1.92	0.67	0.48	‘‘
13	PLC51	CCATGATGAGCCTTGAATGA TCTTCAATCTCCAGGAACACTTT	62	0.23	1.95	2	1.29	0.38	0.22	‘‘
14	PLC60	TGCTTGGACCCTAAATTTGC AAGAAAAGGGCAACCACTGA	60	0.45	1.35	4	1.2	0.4	0.16	‘‘
15	PLC64	ATTGGTGGGGAGTTTGAGTG AAACAACTCATGATGTGCCCT	61	0.49	2.09	3	2.04	0.83	0.51	‘‘
16	PLC66	ATTTGGAGCAAAGATGCAGG GGATCGACCTCCAATCAAGA	60	0.07	2.02	2	1.08	0.17	0.08	‘‘
17	PLC70	CATCTCTTCGTGGCGTAAT AGCAAACAACAGCACACATA	60	0.22	2.19	2	1.46	0.5	0.32	‘‘
18	PLC74	GATTTACCGATGGATCTTCA CTAAGGGAGAGAAAGAAAAGG	61	0.45	2.09	2	2	0.69	0.5	‘‘
19	PLC80	GCTAACAAACAACACCATGA GCATCTAAGTTCTTCAATCTCC	58	0.06	2.05	2	1.02	0.06	0.02	‘‘
20	PLC81	GGGTAGAGTATTATTGAAGGTGG AGAATCGCTAGTTTAGAGCAAG	60	0.56	2	3	2.27	0.91	0.56	Unpublished
21	PLC88	CCAAAACAAGCACCAGTACAAG TAGAAGACGTTGGAGGAGAAGC	59	0.39	2.02	3	1.63	0.69	0.39	‘‘
22	PLC95	TTCATTCTTGGGCTAGGGA TGCAGATGTGAAATACCTCAGT	59	0.48	1.95	3	1.89	0.78	0.47	‘‘
23	PBALC13	GCAGCAGCATGAGAAAATGA ATTACTCGACGCCCCCTAGT	60	0.32	2.19	4	1.5	0.7	0.34	Kaur *et al*. 2011
24	PBALC18	CGTTGGTGGTGCAGTATTTG CCATAAACAAGTGCAATCCAG	60	0.56	2.14	3	2.53	1	0.61	‘‘
25	PBALC24	CCAGAAACATAGAATACTATCACAAGA GCGTCGCAATCACAATATAA	60	0.4	2	3	1.66	0.72	0.4	‘‘
26	PBALC29	TATGCCATTGGATGTGGTTG TATTCAGTTTCCGCCAAAGG	60	0.49	1.93	3	1.85	0.74	0.46	‘‘
27	PBALC224	CCACCCACTTACAAGTACAAA TAAATTGGTGGTGGTGAGTAA	60	0.63	1.95	3	1.44	0.58	0.3	‘‘
28	PBALC250	TGCATTTACCATCATCTCTAAC TGATTGATTCGGTACTTTTTG	60	0.39	1.98	2	1.23	0.34	0.19	‘‘
29	PBALC0260	GTGAACTACCTCTGTGAATGC AGGCGAAATTTCATCTTCTA	60	0.3	2	3	1.67	0.69	0.4	‘‘
30	PBALC0347	CAAAAATGGCTACTTTGATTG GCTTCAGATCAACTGTCTCAG	59	0.38	2.07	3	2.64	1.03	0.62	‘‘
31	PBALC0353	CCATAACAGACAAAACCCTACT ATTCTCAAAGCCCATTTAGTT	59	0.19	2	3	1.58	0.68	0.37	‘‘
32	GLLC 106	ACGACAATCCTCCACCTGAC AACAAGGAAGGGGAGAGGAG	56	0.59	2.12	3	1.67	0.7	0.4	Saha *et al*. 2010
33	GLLC 108	CGACAATCCTCCACCTGAC ACAAGGAAGGGGAGAGGAAG	56	0.69	1.93	3	1.31	0.46	0.24	‘‘
34	GLLC 511	ATTGAGAGGAGGCGGAGAA CGCGTGTCTCTCTCTCTCAC	56	0.62	1.98	4	2.35	1.04	0.58	‘‘
35	GLLC 527	GTGGGACGGTTTGAATTTGA GAACATAAAATGGGAGTGTCACAA	56	0.6	2.05	3	1.52	0.64	0.35	‘‘
36	GLLC 538	AAGGGAAGGAAAAGGGAAGT GCACGAAGAGGGTACGTAGG	56	0.77	2.21	3	2.33	0.93	0.57	‘‘
37	GLLC 541	TGGGCTCATTGAACCAAAAG CCCCCTTTTAAGTGATTTTCC	56	0.52	2.23	2	1.64	0.58	0.39	‘‘
38	GLLC 563	ATGGGCTCATTGAACAAAAG CCCCCTCTAAGAGATTTTCCTC	56	0.48	2.14	5	4.81	1.59	0.8	‘‘
39	GLLC 598	TGGGCTCATTGAACCAAAAG CCCCCTTCTAAGTGATTTTCC	55	0.68	2.19	4	3.25	1.26	0.7	‘‘
40	GLLC 609	GCGACATGGAATTGGATTTG GCACAAAGTCGAGGAGCCTA	55	0.41	1.95	3	2.04	0.83	0.51	‘‘
41	GLLC 614	AACCCCAGCCAGATCTTACA AAGGGTGGTTTTGGTCCTATG	55	0.52	1.98	3	2.01	0.74	0.51	‘‘
42	SSR 124	GAACATATCCAATTATCATC GTATGTGACTGTATGCTTC	52	0.69	1.93	3	2.95	1.09	0.66	Hamweih *et al*. 2005
43	SSR 154	GGAGCAAGAAGAAGCAG GGAATTTATCACACTATCTC	51	0.41	1.95	3	1.69	0.72	0.41	‘‘

Tm = Annealing temperature, PIC = Polymorphism information content, Rp = Resolving power, Na = number of alleles, Ne = genetic diversity, I = Shannons index and He = Nei’s genetic diversity

### Genetic diversity analysis

**Fig 3:**
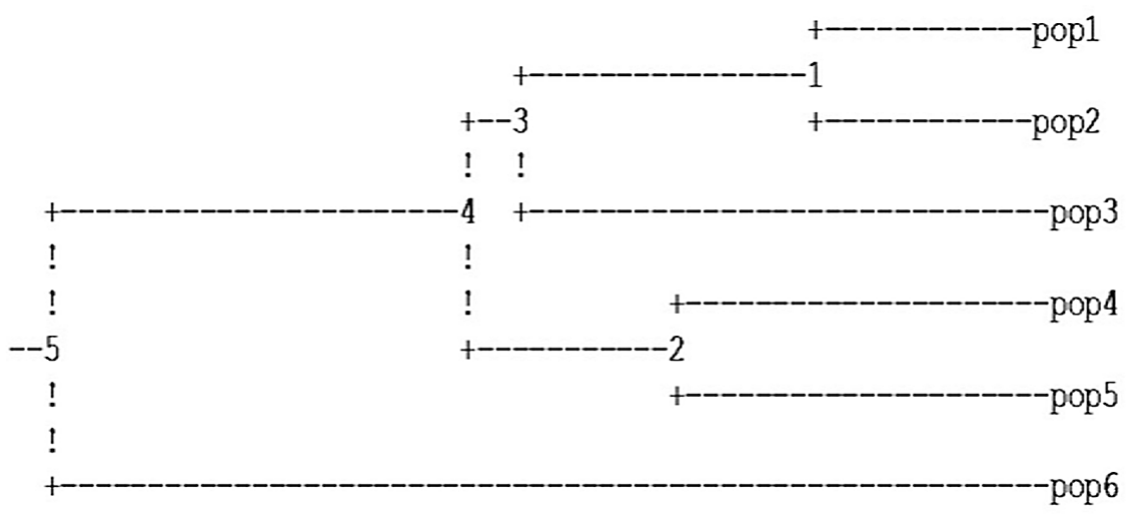
UPGMA Dendrogram based on Nei’s genetic distance using POPGENE version1.31 showing genetic relationship of lentil genotypes among populations.

The Nei’s genetic distance between the subspecies/species ranged from 0.1462 to 0.7143 (Table 3). Pair wise genetic similarities were higher between *L*. *culinaris* subsp. *orientalis* and *L*. *culinaris* subsp. *culinaris* and less between species *L*. *nigricans* and *L*. *culinaris* subsp. *tomentosus*. Cluster analysis of three species based on Nei’s genetic distance also revealed greater similarity between subspecies *L*. *culinaris* and *L*. *orientalis* (Fig 3). *L*. *culinaris* subsp. *odemensis* and *L*. *ervoides* were closer to each other while *L*. *culinaris* subsp. *tomentosus* appeared to be the most distinct among all and grouped separately. Furthermore, analysis of molecular variance revealed that most of the observed genetic variability was among the individuals within subspecies / species (53.8%) than among the subspecies/species(40.76%) (Table 4). Variation within the subspecies/species accounted for 5.44 percent of the total variation. The summary statistics of genetic diversity parameters of different subspecies/species has been mentioned in Table 5. Based on Na, Ne, I and He.*L*. *culinaris* subsp. *orientalis* the progenitor of cultivated lentil *L*. *culinaris* subsp. *culinaris* exhibited maximum diversity among the studied species.

**Table 3:** Nei’s unbiased measures of genetic distance among different subspecies/species of genus *Lens*.

Subspecies / species	*L*. *culinaris* subsp. *culinaris*	*L*. *culinaris* subsp. *orientalis*	*L*. *nigricans*	*L*. *culinaris* subsp. *odemensis*	*L*. *ervoides*	*L*. *culinaris* subsp. *tomentosus*
*L*. *culinaris* subsp. *culinaris*	0	0.864	0.7148	0.6949	0.6995	0.5244
*L*. *culinaris* subsp. *orientalis*	0.1462	0	0.7144	0.6759	0.6995	0.5244
*L*. *nigricans*	0.3357	0.3364	0	0.684	0.6706	0.5237
*L*. *culinaris* subsp. *odemensis*	0.364	0.3917	0.3798	0	0.7181	0.4895
*L*. *ervoides*	0.3574	0.3996	0.3311	0.2364	0	0.5809
*L*. *culinaris* subsp. *tomentosus*	0.6456	0.6469	0.7143	0.6515	0.5431	0

**Table 4:** Analysis of molecular variance (AMOVA) of genus *Lens*.

Source of variation	df	Sum of squares	Variance components	Percentage of variations
Among genus *Lens*	5	576.135	4.05453 va	40.76
Among individuals within subspecies / species	80	899.4	5.3509 vb	53.8
Within subspecies / species	86	46.5	0.5407 vc	5.44
	171	1522.035	9.94613	

**Table 5:** Summary statistics of genetic diversity parameters of genus *Lens*.

Populations	Na	Ne	I	He
*L*. *culinaris* subsp. *culinaris*	2.046	1.455	0.405	0.2090
*L*. *culinaris* subsp. *orientalis*	2.511	1.549	0.522	0.2090
*L*. *nigricans*	1.604	1.283	0.279	0.2090
*L*. *culinaris* subsp. *odemensis*	1.604	1.357	0.313	0.2090
*L*. *ervoides*	2.023	1.431	0.421	0.2090
*L*. *culinaris* subsp. *tomentosus*	1.139	1.102	0.084	0.2090

Na- Number of alleles; Ne-Number of effective alleles; I- Shannon index; He-Expected heterozygosity

**Fig 4:**
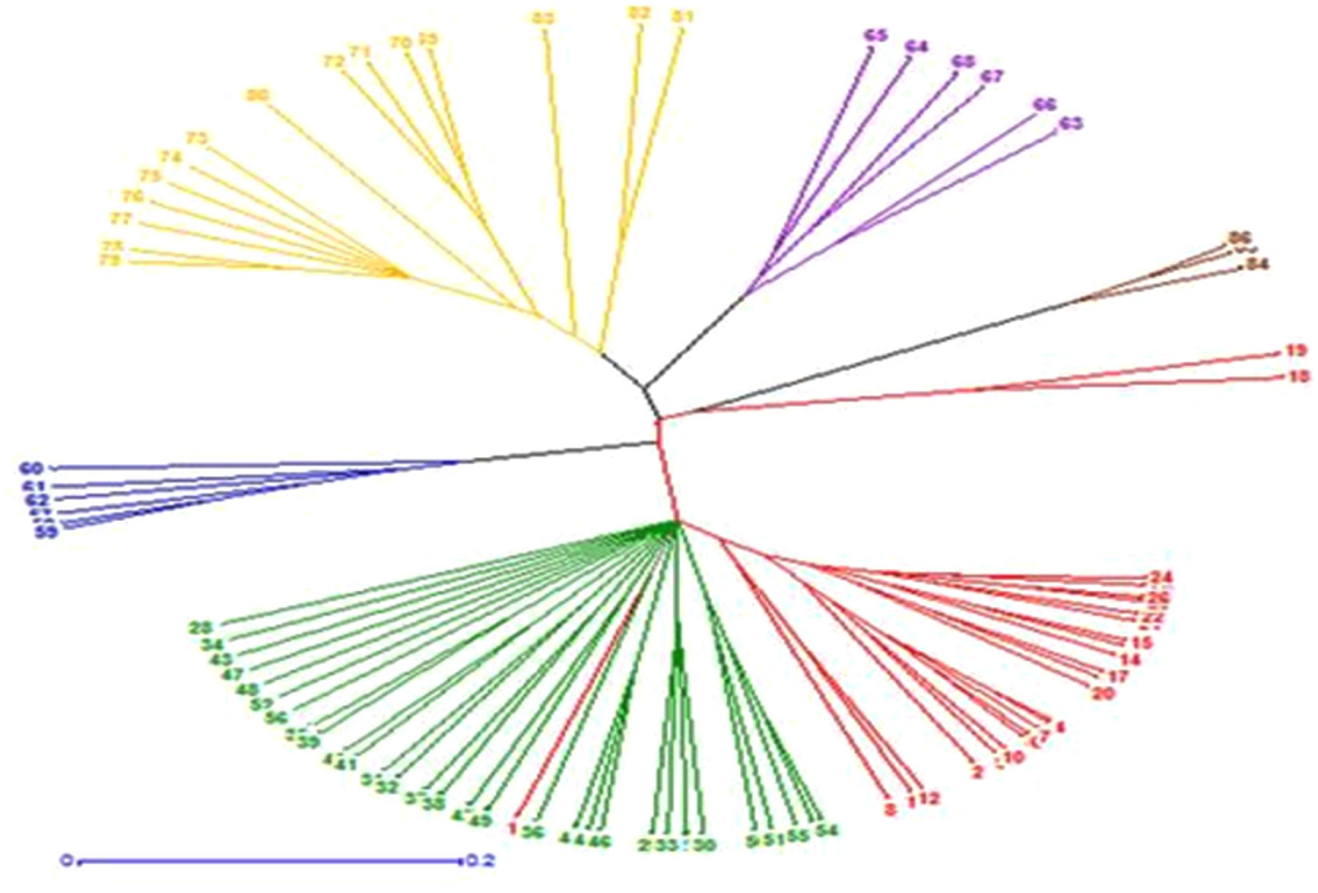
Genetic relationship among 86 *lens* accessions using Unbiased neighbouring joining dendrogram of 43 microsatellite loci. Red colored—*L*. *culinaris* subsp. *culinaris* (1–26), Green colored-*L*. *culinaris* subsp. *orientalis* (27–56), Blue colored—*L*. *nigricans* (57–62), Purple colored—*L*. *culinaris* subsp. *odemensis* (63–68), Yellow colored—*L*. *ervoides* (69–83), Brown colored—*L*. *culinaris* subsp. *tomentosus* (84–86).

The unweighted neighbor-joining (UNJ) dendrogram constructed on the basis of genetic similarity matrix grouped the 86 genotypes into six clusters *viz*., (Fig 4). The unweighted neighbour-joining (UNJ) dendrogram constructed on the basis of genetic similarity matrix grouped the 86 *Lens* genotypes into six clusters. Cluster 1 comprised of *L*. *culinaris* subsp. *culinaris* genotypes no. 1to 26 (except genotype no 18 and 19). Cluster II comprised of *L*. *culinaris* subsp. *orientalis* genotypes *no*. 27–56. Cluster III included *L*. *nigricans* genotypes *no*.57-62. Cluster IV comprised of *L*. *culinaris* subsp. *odemensis* genotypes *no*.63-68. Cluster V consisted *L*. *ervoides* genotypes no. 69–83. Cluster VI consisted *L*. *culinaris* subsp. *tomentosus* genotypes *no*.84-86 and two *L*. *culinaris* subsp. *culnaris* genotypes no. 18 and 19.

### Population structure and genetic relationships among genotypes

**Fig 5:**
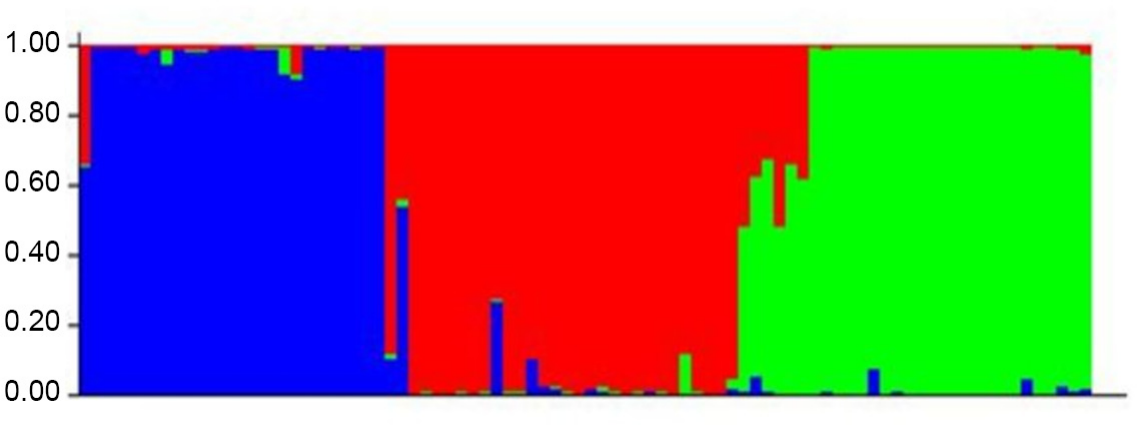
STURUCTURE analysis of genus *Lens* based on EST and Genomic SSR.

SSR allelic diversity data was used to estimate Pritchard’s structure of 86 genotypes at k = 3. The best goodness of fit was found at k = 3, on the basis of estimated posterior probability of data. The inferred ancestry of the genotypes allocated all cultivars to three major clusters belonging to three species (Fig 5). The population structure clearly distinguished various species. The first inferred cluster includes 25 *L*. *culinaris* subsp. *culinaris* genotypes with more than 90 percent co-ancestry values, one *L*. *culinaris* subsp. *culinaris* genotype with 65% similarity and one *L*. *culinaris* subsp. *orientalis* genotype IG-135443 sharing 53% similarity with *L*. *culinaris* subsp. *culinaris*. The second group exclusively comprised of 28 *L*. *culinaris* subsp. *orientalis* genotypes with more than 85 percent inferred ancestry, one genotype with 72.6% and two *L*. *nigricans* genotypes sharing more than 50 percent co-ancestry with *L*. *culinaris* subsp. *orientalis* genotypes. The third inferred cluster comprised of the remaining four wild type subspecies / species represented by a single inferred cluster exhibiting common ancestry among the subspecies / species. It includes four *L*. *nigricans* genotypes, six *L*. *culinaris* subsp. *odemensis*, fifteen *L*. *ervoides* and three *L*. *culinaris* subsp. *tomentosus* genotypes. The least allele frequency divergence among population structure was observed between first and third inferred cluster (0.1073). The population structure revealed maximum expected heterozygosity between individuals in the same cluster for the second inferred cluster (0.367).

## Discussion

In the present study, we investigated the genetic diversity and population structure among three *Lens* species using EST-SSR and Genomic SSRs. The total number of alleles detected by the 43 SSR loci was highest in *L*. *culinaris* subsp. *orientalis* followed by *L*. *culinaris* subsp. *culinaris* and other four wild subspecies/species which is in accordance to that observed by Hamweih *et al*. [28] our data also suggests that the genetic diversity is greater in wild species as compared to the cultivated *L*. *culinaris* subsp. *culinaris* genotypes. Similar findings have been reported by Choudhary *et al*. [53] in genus *Cicer*.

We observed that EST-SSRs despite having advantage in ease of development and higher cross-species transferability rate provided polymorphism at low levels as compared to genomic SSRs. Genomic SSRs exhibited higher average Rp and PIC value in comparison to EST SSRs. EST-SSRs are developed from regions of the genome associated with a trait of interest and could be part of the gene controlling the character [36]. The functional markers exhibit transferability as the genic regions are more conserved, as compared to anonymous ones [32]. Higher gene diversity and more effective number of alleles were revealed by genomic SSRs. Hu *et al*. [54] also reported that genomic SSRs revealed more polymorphism than EST-SSR in cucumber species. This might be explained due to greater variation in SSR flanking regions in the non-coding regions due to selection pressures compared to the coding regions. According to Hu *et al*. [54], estimation of genetic diversity would be more effective with combination of both genomic and EST-SSRs in plant species with narrow genetic base. Hence a combination of genomic SSR and EST-SSR was used to assess the genetic diversity in the present study.

### Comparison of EST-SSR and genomic SSR markers

To estimate genetic variability in crop improvement research, increasingly crop specific microsatellite markers were used. The precise study of genetic diversity can be done by using microsatellite markers from both coding and non-coding regions of the genome. Therefore we have included microsatellite markers located in both genic and non-genic regions to study diversity among the three *Lens* species. Genomic SSRs were more polymorphic in wild species compared to the cultivated ones while the reverse was observed for EST-SSRs (S2 Fig). Species *L*. *ervoides* produced highest number of alleles with genomic SSRs while subspecies *L*. *culinaris* subsp. *orientalis* produced the highest number of alleles with EST-SSRs (S3 Fig).

Twenty two EST SSRs (PLC series Table 2) developed in our lab (using protocol suggested by Jain *et al*. [31] exhibiting polymorphism comprised of six mono, di-, tri- nucleotides motif each and one penta-, one hexa- nucleotide motif. Trinucleotide repeats were obtained in greater frequency in a number of crop species such as sugarcane [55], foxtail millet [43] [56], *B*. *rapa* [57], barley [36], chickpea [58] and Phyllostachys [59]. The greater frequency of trinucleotide motifs explained their better accommodation in reading frames without disturbing the overall sequence [60]. Development of large number of EST SSRs with specific motifs is required for establishing relationship between SSR motifs (type and size) and polymorphism level in lentil.

### Analysis of genetic diversity in different *Lens* species

The cluster analysis revealed that most of the cultivated *L*. *culinaris* subsp. *culinaris* genotypes grouped with the wild *L*. *culinaris* subsp. *orientalis* genotypes. The other wild species were scattered into different groups. Our observation is in concurrence to that reported by Hamweih *et al*. [28] where *L*. *odemenesis* subsp. *culinaris* and *L*. *culinaris* subsp. *tomentosus* grouped together while the other two subspecies, *L*. *culinaris* subsp. *orientalis* were found closer to each other. The progenitor of cultivated lentil *L*. *culinaris* subsp. *culinaris* is *L*. *culinaris* subsp. *orientalis*.

Population structure analysis revealed three species among the *Lens* accessions. The three species without assuming any population structure grouped *L*. *culinaris* subsp. *culinaris*, *L*. *culinaris* subsp. *orientalis* and the rest four wild subspecies/species into three precise distinct clusters. Population structure obtained after assuming prior populations revealed only two major inferred clusters at optimum delta k value. Both the *L*. *culinaris* subsp. *orientalis* and *L*. *culinaris* subsp. *culinaris* formed one inferred cluster and the rest four wild subspecies/species formed another inferred cluster. *L*. *culinaris* subsp. *culinaris* appeared close to wild subspecies *L*. *culinaris* subsp. *orientalis* compared to other four wild subspecies / species. Previous study also reported the *L*. *culinaris* subsp. *culinaris* and *L*. *culinaris* subsp. *orientalis* are quite distinct from other wild subspecies [28]. In our study, the highest genetic divergence was observed among *L*. *culinaris* subsp. *orientalis* which is similar to that reported earlier in *Lens* species [20]. In our study three accessions of *L*. *tomemtosus* subsp. *culinaris* were grouped together with two accessions of *L*. *culinaris* subsp. *culinaris* in cluster VI. The report is in agreement with earlier study on classification and characterization of species within genus *Lens* using genotype by sequencing [61]. In this study *L*. *culinaris* subsp. *culinaris*, *L*. *culinaris* subsp. *orientalis* and *L*. *tomemtosus* subsp. *culinaris* were grouped together as primary gene pool.

Pure-line selection from land races was initial breeding method. Later the hybridization efforts were made in late 1970’s. Few improved land races were used repeatedly in the hybridization programme. Adequate genetic gains were not recorded and productivity remained low due to loss of genes for higher productivity and resistance to biotic and abiotic stresses. The reasons for low productivity are narrow genetic base of Indigenous *microsperma* germplasm (i.e. *pilosae* type), repeated use of few genotypes in breeding programs [28] and susceptibility to biotic and abiotic stresses. The molecular diversity analysis has revealed the narrow genetic base of released varieties and germplasm lines [62–63]. Therefore for broadening the genetic base of lentil in South Asia introgression of the alien genes from the exotic materials and related wild species was suggested [6–9]. The Mediterranean lentil germplasm is characterized by long duration and bold seed size. The differences in flowering duration restricted their use in breeding programme in South Asia. Precoz an introduction from ICARDA was identified as source of earliness, bold seed size and rust resistance [8] [64] [65] and was extensively used in breeding programmes. Recently ICARDA germplasm line ILL 6002 was identified as source of early vigour and bold seed size. The studies by Gupta *et al*. [66] and Singh *et al*.[67] indicated the utility of wild species for agronomic traits and wild species. Recently Singh *et al*. [68] reported the potential of global wild species for broadening the genetic base and yield improvement. *L*. *nigricans* exhibited potential for improvement of yield traits and *L*. *ervoides* for biotic stresses. Substantial gains in productivity can be achieved by utilization of wild species and Mediterranean germplasm lines. However to maintain the photoperiod sensitive wild species and Mediterranean land races artificially lengthened days in the greenhouse are required. In summary, EST-SSR are promising molecular resources for germplasm characterization. Though the polymorphism exhibited by EST-SSRs is low in comparison to genomic SSRs. The diversity analysis EST-SSR and genomic SSR revealed the potential for use of related wild of genus *Lens* for broadening of genetic base of cultivated lentil.

For construction of high-density genetic linkage map, comparative mapping, evolutionary studies and identification and mapping of genes/quantitative trait loci (QTLs) for useful agronomic traits large number of genome wide microsatellite markers are required [56][58]. The development of high-throughput genome wide markers requires Next-Generation sequencing (NGS). These techniques permits millions of bases to be sequenced in one round, at a very low cost as compared to traditional Sanger sequencing [69][45].

## Supporting Information

S1 Fig A representative gel picture of *Lens* species with marker GLLC609 (for decoding of the numbers refer S3 Table). (TIFF)

S2 Fig Variation in PIC value of genus *Lens* with EST and Genomic SSR. (TIFF)

S3 Fig Variation in No. of alleles (Na) of genus *Lens* with EST-SSR and Genomic SSR. (TIFF)

S1 Table A table containing [i] marker ID, [ii] number of alleles obtained, [iii] range of alleles amplified (bp), [iv] size(s) obtained in accessions (1 to 86). (XLSX)

S2 Table The primer sequences deposited in NCBI probe database PUIDs and motifs of the developed markers. (XLSX)

S3 Table Eighty six accessions of different *Lens* species used in the study. (XLSX)
