# Supplementary figures and images for: Genetic Diversity in Lens Species Revealed by EST and Genomic Simple Sequence Repeat Analysis
Source: PLoS One. 2015 Sep 18;10(9):e0138101. doi: 10.1371/journal.pone.0138101 (PMC4575128; doi:10.1371/journal.pone.0138101)

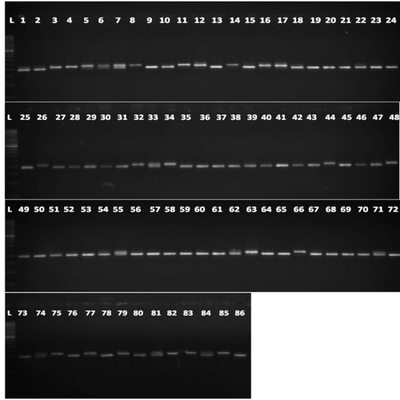

Supplement: S1 Fig — (TIFF) [file pone.0138101.s001.tiff]

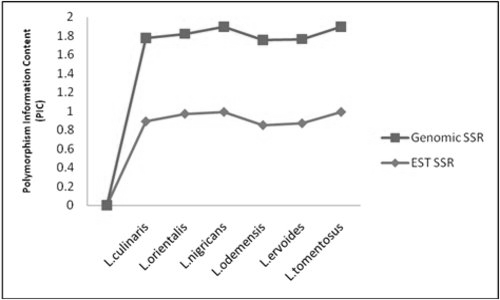

Supplement: S2 Fig — (TIFF) [file pone.0138101.s002.tiff]

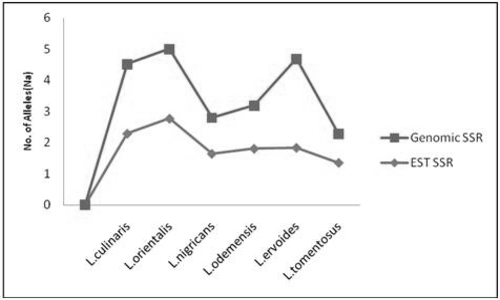

Supplement: S3 Fig — (TIFF) [file pone.0138101.s003.tiff]
